# Technologies to Support Assessment of Movement During Video Consultations: Exploratory Study

**DOI:** 10.2196/30233

**Published:** 2021-09-24

**Authors:** Ray B Jones, Suzanne Hubble, Lloyd Taylor, Hilary Gunn, Angela Logan, Tim Rowland, Hannah Bradwell, Luke J Connolly, Kim Algie, Krithika Anil, Bradley Halliday, Sandra Houston, Rachel Dennett, Sarah Chatfield, Sarah Buckingham, Jennifer Freeman

**Affiliations:** 1 Centre for Health Technology University of Plymouth Plymouth United Kingdom; 2 School of Health Professions University of Plymouth Plymouth United Kingdom; 3 Royal Devon and Exeter NHS Foundation Trust Exeter United Kingdom; 4 Royal Cornwall Hospital Trust Truro United Kingdom; 5 Liskeard Community Hospital Liskeard United Kingdom; 6 Mount Gould Hospital Plymouth United Kingdom

**Keywords:** tele-rehabilitation, video-consultations, assessment of movement, eHealth, technology, desktop robots, wide-angle webcams, physical health, rehabilitation, remote, assessment, assistive technology, evaluation, framework, webcam, telehealth, robots

## Abstract

**Background:**

Understanding and assessing patients’ body movements is essential for physical rehabilitation but is challenging in video consultations, as clinicians are frequently unable to see the whole patient or observe the patient as they perform specific movements.

**Objective:**

The objective of this exploratory study was to assess the use of readily available technologies that would enable remote assessment of patient movement as part of a video consultation.

**Methods:**

We reviewed the literature and available technologies and chose four technologies (Kubi and Pivo desktop robots, Facebook Portal TV, wide-angle webcam), in addition to help from a friend or a simple mobile phone holder, to assist video consultations. We used 5 standard assessments (sit-to-stand, timed “Up & Go,” Berg Balance Test, ankle range of motion, shoulder range of motion) as the “challenge” for the technology. We developed an evaluation framework of 6 items: efficacy, cost, delivery, patient setup, clinician training and guidance, and safety. The coauthors, including 10 physiotherapists, then took the roles of clinician and patient to explore 7 combinations of 5 technologies. Subsequently, we applied our findings to hypothetical patients based on the researchers’ family members and clinical experience.

**Results:**

Kubi, which allowed the clinician to remotely control the patient’s device, was useful for repositioning the tablet camera to gain a better view of the patient’s body parts but not for tracking movement. Facebook Portal TV was useful, but only for upper body movement, as it functions based on face tracking. Both Pivo, with automated full body tracking using a mobile phone, and the wide-angle webcam for a laptop or desktop computer show promise. Simple solutions such as having a friend operate a mobile phone and use of a mobile phone holder also have potential. The setup of these technologies will require better instructions than are currently available from suppliers, and successful use will depend on the technology readiness of patients and, to some degree, of clinicians.

**Conclusions:**

Technologies that may enable clinicians to assess movement remotely as part of video consultations depend on the interplay of technology readiness, the patient’s clinical conditions, and social support. The most promising off-the-shelf approaches seem to be use of wide-angle webcams, Pivo, help from a friend, or a simple mobile phone holder. Collaborative work between patients and clinicians is needed to develop and trial technological solutions to support video consultations assessing movement.

## Introduction

The COVID-19 pandemic has focused attention on remote consultations, and although there is evidence supporting the feasibility and acceptability of telephone and video-based rehabilitation for patients and practitioners [1,2], challenges remain. Relatively little work has been published on the remote assessment of movement as needed in the rehabilitation of people with a physical disability, including those recovering from COVID-19. Understanding and assessing patients’ body movements is essential for physical rehabilitation but is challenging in video consultations, as clinicians can only see the patient on a 2D screen; thus, they are frequently unable to see the whole patient or see the patient performing specific movements or functional activities. Although anecdotally, various technologies may have been discussed, there is little advice available for clinicians to address this issue. A recent review ([3], forthcoming) included 11 primary studies, 3 reviews, and 9 guidance documents, and it was noted that (1) telerehabilitation guidance was not specific to movement-related assessment and (2) most research studies provided neither guidance nor training of movement-specific assessment to practitioners.

In our recent survey of 247 UK-based health [4] and social care practitioners, over half of those who carried out video consultations for movement assessments [4] reported concerns regarding the validity and reliability of remote physical assessments. Central to these concerns were technology-related issues (including poor internet connections and hardware issues, resulting in poor audio and visual quality) and physical examination restrictions, including a limited view of the patient, not being able to “feel” movement, and difficulty gaining an accurate assessment of the many aspects of mobility (eg, range, velocity, quality, endurance) that are important in rehabilitation. One concern for many respondents, specific to video consultations that assess movement, was difficulties positioning the camera. For example, one physiotherapist in the field of neurology said, “The camera angle does not give you a true image of the range of movement.” Ensuring a good field of view was perceived as centrally important for a successful video consultation. A consultant in rehabilitation medicine said, “My top three tips? Position of camera, position of camera, position of camera!” Difficulties with camera angles, limited field of view, and tracking movement are common obstacles experienced by clinicians working in telehealth [5,6].

Video consultations are typically undertaken with clinicians using a laptop and patients using either a laptop, tablet, or mobile phone via software such as Attend Anywhere [7]. Telepresence robots, videocall technologies embedded in robots controlled by the caller to give the sense of “being there,” have often been suggested as the future direction for remote home care, and there has been considerable investment in their development and evaluation [8,9]. Although the cost of commercial telepresence robots has decreased considerably over recent years (eg, Giraff cost £5000 [US $6940] in 2013, while Padbot cost £900 [US $1249] in 2020), as of March 2021, they were not yet ubiquitous or affordable for mass use in telerehabilitation. However, much of the sophistication and hence the cost of telepresence robots lies in their motor and guidance capabilities. Therefore, we postulated that desktop robotics in which the camera on the device can be rotated or angled to follow movement might be sufficiently affordable, effective, and feasible to use remotely, such as when required during a pandemic lockdown.

We were aware of two potential desktop robot devices, Kubi and Pivo. To check for other suitable technologies or approaches, we reviewed the literature, searching three bibliographic databases (Web of Science, MEDLINE, and CINAHL) for published literature from 2017 (Multimedia Appendix 1). We identified two papers [10,11] of relevance.

Wu et al [10] investigated the usability of the Kubi desktop telepresence robot in older people with self-reported mobility impairments. They studied 5 people and reported that the Kubi movement speed, controls, and user interface were a limitation of this device. This work was published in 2017; therefore, we thought Kubi warranted further inclusion in our investigations. However, we had also identified a newer and less expensive but similar device: Pivo. We therefore included Kubi and Pivo (£600 [US $833] and £85 [US $118], respectively; March 2021) (Multimedia Appendix 2) as devices that could potentially track a patient’s movement. The manufacturers of Kubi describe it as “desktop robotics” (Table 1). Currently (March 2021), Kubi allows the clinician to remotely control the position of a tablet using an interface on their tablet or laptop (Table 1). The Pivo Pod is a small cylindrical and wireless device, and it could equally be called a “desktop robot.” It is approximately 3 inches tall, with a mount attached to the top that can hold a smartphone and rotate 360 degrees, automatically following the user (either their head or whole body). The smartphone (both IOS and Android) requires the Pivo Meet app and uses Bluetooth to pair the Pivo Pod to the phone. Pivo Meet is a 1-1 video chat application that supports video consultations, during which the automatic tracking of the Pod will follow any movement.

**Table 1:** 7 new permutations of the 5 technologies assessed.

Technology	Permutations	Image
Kubi Plus	This desktop robotic device can be remotely controlled by the clinician during the appointment; the setup also includes a 10-inch tablet computer (Lenovo Group Limited).	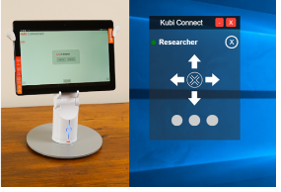
Pivo	Using Pivo Pod software, this device tracks the patient around a room. The patient records and sends the video. Using Pivo Meet software, the same procedure as above is performed, but in real time during a video call.	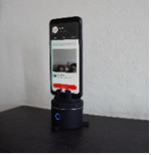
Wide-angle webcam	We tested the Brio Stream Webcam (Logitech International SA), but we also include a brief review of other possible devices in Multimedia Appendix 3.	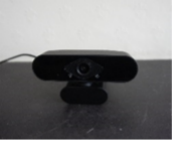
Facebook Portal TV	This device, with millions of users globally, includes a wide-angle webcam with software that tracks the user around the room (to some degree). It uses Facebook Messenger or WhatsApp video (owned by Facebook).	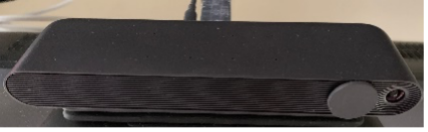
Mobile phone	The mobile phone (eg, iPhone) is operated by a friend. The mobile phone is operated by the patient but with use of a stand.	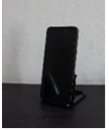

In considering devices (Kubi and Pivo) that moved to track patients’ movement, webcams that could automatically track participants or had sufficiently wide angles so that participants could be seen at all times seemed relevant to consider. Venkateraman et al [11] studied gait in 42 ambulant veterans, evaluating the reliability and validity of the Tinetti Performance-Oriented Mobility Assessment gait scale (POMA-G) using a single fixed laptop or tablet camera [11]. Recorded video footage of patients conducting the assessment was compared to in-person assessments, and no significant differences were found in reliability and validity between video assessments and in-person POMA-G assessments. However, it was necessary to have both front and lateral views of the patient. Therefore, we also tested a generic wide-angle webcam and Facebook Portal TV (which includes a tracking webcam).

In summary, the aim of this exploratory study was to assess technology-supported methods for video consultations in which movement is assessed. We assessed Kubi, Pivo, Facebook Portal TV, and a wide-angle webcam, as well as help from a friend or family member with a mobile phone or a simple holder for a mobile phone, for their potential to undertake a video consultation assessing movement.

## Methods

### Ethics

Ethical permission was neither needed nor sought. All trials were conducted by the co-authors, who acted as either the clinician or the simulated patient.

### Assessment Challenge

While recognizing the complexity of movement as a construct, we needed a “typical” physical assessment challenge that might be experienced within a video consultation. We based this challenge on 5 standardized and validated physical tests, which we selected because they are commonly used measures within the face-to-face rehabilitation environment: (1) sit-to-stand in 30 seconds [12], (2) timed “Up & Go” test [13], (3) Berg Balance Test [14], (4) visual estimation of ankle range of motion [15], and (5) visual estimation of shoulder range of motion [16] (Multimedia Appendix 4).

### Assessment Framework

The first criterion was feasibility and efficacy; could the clinician complete the assessment challenge using this equipment? This was then extended, and an assessment framework was developed using ideas from the work of Tyson and Connell [17]. They noted that although there were many tools that measured mobility, nearly all had been developed for use in research and were impractical or inadequately developed for everyday clinical use. Their systematic review recommends the best measures to use with neurological and stroke patients in the clinical setting. They developed criteria and a scoring system for clinical utility based on four questions: (1) What is the time taken to administer, analyze, and interpret the measure? (2) What is the cost? (3) Does the measure need specialist equipment and training to use? (4) Is the measure portable?

We built on these four ideas for video consultations involving assessment of movement. “Time” evolved to become (1) the elapsed time to send equipment to the patient, (2) the time and setup process required of the patient and/or their family member or friend, (3) the time for the clinician to set up the equipment and become experienced in using it (initial setup) and then to set up for each subsequent patient. “Cost” was divided into (1) capital cost for equipment (considering life expectancy and obsolescence of equipment) and (2) revenue cost in getting equipment to and from patients. “Training” was combined with usability, as in, “How difficult is this technology to use for clinicians? Do they need guidance or training? How long would it take clinicians to get set up for each patient once they were familiar with the equipment?” “Portability” was combined with the time and setup process required of patients; this also considered whether patients required their own equipment as part of the setup. Another criterion, perhaps assumed to be “dealt with” in face-to-face consultations, is patient safety. This is important in considering telerehabilitation both from the point of view of physical safety, for example from falling or from infection prevention and control through to data safety, when comparing to face-to-face consultations. Safety was added as a sixth element of the assessment framework.

The assessment framework therefore became:

1. Efficacy: can you carry out the assessment?
2. Capital or licensing cost: what is the current cost of the technology for the National Health Service (NHS)?
3. Delivery: for people with limited mobility and those in rural areas with no nearby post office, and for all during the pandemic, what is the best option? (Courier delivery and collection appeared to be the best option. We cited prices from couriers based on a 30-mile journey for various package sizes.)
4. Patient setup: what are the time and challenges involved for patients in getting the equipment set up and ready for video consultations?
5. Clinician training and guidance: how difficult is the use of this technology for clinicians? Do they need guidance or training? How long will it take clinicians to get set up for each patient once they are familiar with the equipment?
6. Safety: How physically safe are patients when using this equipment at home? How safe are any data that may be transmitted from the point of view of data security and confidentiality?

Finally, Tyson and Connell [17] had a specific patient group in mind for their review; we considered which patient groups might be suitable for different technology scenarios. We discuss these as a whole rather than individually for each technology, and we consider both the patient’s technological readiness and clinical condition.

### Technology Options and Specifications

We examined 7 new permutations of 5 technologies against the 6 criteria of the assessment framework. The 5 technologies were Kubi, Pivo (either for the patient alone on their own time or “live” during video consultation), wide-angle webcam, Facebook Portal TV, and mobile phone (either held by a friend or by the patient on their own using a stand or with no additional hardware) (Table 1). We were aware that technology specifications change rapidly and, for example, use of a mobile phone with a low-specification camera and processor will perform very differently from a “cutting-edge” phone with high-end specifications. Furthermore, broadband and Wi-Fi network speeds may have a major influence on technology performance. We aimed to trial the technologies in a number of settings and to carefully document the technologies used. Full specifications (March 2021) are given in Multimedia Appendix 4.

### Environment

We tested the technologies in a range of environments, including people’s homes with less or more spacious rooms, and in sunlight and artificial light.

### Usability and User Instructions

All technologies came with manufacturer instructions for setup; however, reference to web-based help and user group commentaries as well as help desk user guidance was often also required. However, it is reasonable to posit that clear and easy-to-follow instructions can be written, and we present our results based on the assumption that the technology would be used with clear installation and user guidance.

### Participants

Members of the author team took on the roles of clinician and patient, and they also discussed the use of the technologies with family members. Nine members of the author team were practicing clinicians using remote consultations/ telerehabilitation, and one member was a student clinician. Other coauthor participants were staff members from a center for health technology.

## Results

### Technology Assessment

The baseline assessment was use of a mobile phone and no additional hardware or help. The patient used Attend Anywhere or other video consultation software, and they were required to find a way to balance the phone on a piece of furniture to allow the clinician to see them in full view of the camera. This is possible if the patient is resourceful, is physically capable, and has sufficient space. As with all options using the patient’s mobile phone, a key limitation is poor image quality as a result of Wi-Fi or telephone network availability, lighting, or the quality of the camera on the mobile phone. Safety concerns about the patient’s home space and maneuvering around environmental obstacles while undertaking the requested movements apply to this and all scenarios.

Neither Kubi, Pivo, nor Facebook Portal TV were rated as being easy to set up; all users in this assessment challenge had to seek web-based help and help desk user guidance. Further results (summarized in Table 2) were obtained once the technology was set up. Capital costs are presented at current prices for one item, assuming the NHS must buy and provide the device. Marginal costs would be zero if the patient already owned the device. If the device was NHS owned, it would be used by many patients sequentially over the life of the device. Costs also assume that the patient has Wi-Fi service.

**Table 2:** Summary of findings for the 7 new scenarios and 6 assessment criteria.

Technology/assistance in addition to “normal” video consultation		Assessment criteria					
		Efficacy	NHS^a^ capital cost	NHS delivery cost	Patient setup	Clinician training and guidance	Safety^b^
Kubi + tablet		Good for outcome measures that did not require tracking; tracking poor due to time lag	£437^c^ for Kubi plus £110 for Lenovo tablet	£40	Issues with device not holding charge, on/off button, Wi-Fi connection, instructions	Simple; the clinician calls the patient and can easily go from one patient to the next	Unable to view patient when not tracking; potential to lose sight of loss of balance/falls. Data security not an issue, as this approach involves continued use of standard software
**Pivo**							
	Recorded	Good for all outcome measures; patient must transfer data file	£85	£26	Issues with connection, instructions	Simple	Data security unknown; more exploration needed
	Live	Good for all outcome measures, but patient contacts clinician	£85	£26	Issues with connection, instructions	Patient must call clinician; issues with instructions	Data security unknown; more exploration needed
Wide-angle webcam		Good for all outcome measures, but only works for laptop or personal computer	£190	£26	Only works for laptop or personal computer, but simple	Simple	Data security not an issue, as this approach involves continued use of standard software
Facebook TV Portal		Only works for upper body (feet not in picture); unable to effectively track faster walking; only usable in patient’s TV room	£140	£26	Requires Wi-Fi–connected smart TV; issues with instructions	Simple; the patient can be added to the clinician’s mobile contacts to make a WhatsApp call	Data security—some concerns related to using WhatsApp
**Mobile phone**							
	Friend using mobile phone	Good for all outcome measures if a friend is available	£0	£0	Need to be able to call a friend	Simple	Data security not an issue, as this approach involves continued use of standard software
	Mobile phone holder	Patient may leave field of view during tracking	£26	£26	Simple	Simple	Data security not an issue, as this approach involves continued use of standard software

^a^NHS: National Health Service.

^b^All technologies have safety considerations regarding space and collision with furniture.

^c^1 British pound=US $1.39.

### Kubi

#### Efficacy (Assessment Challenge)

For operation, the system was rated as easy to use, although “a bit clunky”; moreover, clinicians were required to accustom themselves to the “loading bar” movement in relation to the space of the patient's room. Kubi worked well to capture outcome measures that required repositioning or did not require tracking of the patient (eg, opening a conversation in one part of the room, followed by the clinician repositioning the tablet angle when the patient moved to another area for movement assessment or to view body parts, such as feet). It was possible to complete a Berg Balance Test, assess range of movement, and undertake a sit-to-stand test at a distance of >2 m from the Kubi. However, assessing the quality of the movement was more challenging due to the low picture quality/time lag and “jerkiness” of the Kubi image. When tracking (ie, following someone’s walking/movement with rotation of the tablet), the Kubi did not respond quickly, the user interface was cumbersome, and the tracking speed was fixed. As a result, the patient was lost from view, which was problematic for walking and turning assessments. Clinicians were often unable to observe the movement unless the patient positioned the Kubi far enough away to make the whole person visible on the screen; this was challenging when considering environmental constraints such as space and furniture.

#### Patient Setup

An issue was encountered with the batteries in Kubi devices not holding a charge. This led to connection problems, creating confusion during setup when trying to follow instructions on pairing the Kubi devices with partner tablets. Other issues raised were a problematic on/off button and problems connecting to the Wi-Fi network. The authors who trialed Kubi thought that despite being “tech savvy” and having the manufacturer’s instructions, they needed numerous “work-arounds” and much time to set up. It is unclear if well-written instructions and instructional videos would overcome this problem.

#### Clinician Training and Guidance

Clinician setup of the software on their laptop was relatively simple. In clinical practice, when dealing with a number of patients, the software would typically be loaded and “ready to go” on the clinician’s laptop or desktop computer. Although we tested Kubi using Zoom, it could be used with Attend Anywhere or other video consultation software. The clinician could move between patients quickly with the next patient’s Kubi ID number and Attend Anywhere link.

#### Safety

The inability to track patients effectively raised safety concerns; clinicians could lose sight of walking patients who were becoming unsteady or falling, and the clinicians were thus unable to provide instructions or prevent the fall. There were particular challenges when the physical environment involved restricted space, as patients inevitably needed to move closer to the camera, thereby preventing the clinician from seeing the whole person. Data security with Kubi is good; it allows the user to run NHS-approved software such as Attend Anywhere and therefore does not have the data security concerns of some other technologies.

### Pivo (Recorded)

#### Efficacy (Assessment Challenge)

Pivo Pod allows for either facial or head-to-toe artificial intelligence (AI) tracking. With tracking speeds from slow to “frenzy,” the Pivo easily tracks the patient’s movements from side to side. The Pivo also automatically zooms and focuses during the video. All 5 assessment challenges were achieved with this device. The Pivo would be a valuable tool for recording short video clips in the home environment, such as standing up and moving from a chair or wheelchair, lifting and carrying objects, impact of fatigue through the day, and gait in the home environment.

#### Patient Setup

If the Pivo is sent complete with a mobile phone, it is necessary to connect the Pivo to a Wi-Fi or mobile network. If the patient uses their own mobile phone, they will need to download and install software via the app and sign in via an email or Pivo account. There are a number of Pivo apps, which creates potential for confusion. Depending on the patient’s technical literacy, they may require assistance with the initial video operation and selection of features, such as AI tracking. Transferring the file may also be challenging. A 2-minute video is 225 MB in size, which is too large for most email servers; thus, an alternative file sharing platform is required, which adds complications for the patient.

#### Clinician Training and Guidance

The clinician needs to access the video files from a file sharing platform; however, this is time-efficient for clinicians, as they can go from one patient file to another.

### Pivo (Live)

#### Efficacy (Assessment Challenge)

In Pivo Meet (live call), clinicians were able to complete all assessments. Patients remained in view of the automatic tracking, with 2 m distance from the camera required for full body view. Auto-tracking was better for side-to-side movements than for forward-and-back movements. In addition to auto-tracking, clinicians could control the movement of the Pivo. Tracking was responsive and smooth; however, vertical (up-down) adjustments to the camera angle could not be made.

#### Patient Setup

Physical setup simply involved placing a phone onto the Pivo holder. Downloading and setting up the Pivo Meet app was more difficult, but it should be possible to simplify this process.

#### Clinician Training and Guidance

The clinician cannot initiate the consultation and must wait for the patient to send them a call link; therefore, for efficient use of clinician time, a health care assistant or an administrator should perhaps receive the Pivo call and keep the patient waiting for the clinician.

### Wide-angle Webcam

#### Efficacy (Assessment Challenge)

A wide-angle webcam proved to be a simple solution, provided there was sufficient room (at least 3 m from the camera) to allow a full-body view. In one trial, there were some problems with lighting in the patient’s home; however, this could occur with any device. In a room where overhead lighting or lighting behind the camera was possible and there were no environmental obstacles, the patient’s movements and actions were fully visible at all times, and it was possible to effectively complete all 5 outcome measures. Care may be needed in choosing webcams with automatic light adjusting software.

#### Patient Setup

In theory, setup of a webcam should be “plug and play”; however, in practice, further checks are needed. This is only relevant for patients who have laptop computers, desktop computers, or a device that requires an additional webcam. It is not applicable for patients who only have tablets or mobile phones.

### Facebook Portal TV

#### Efficacy (Assessment Challenge)

Users reported no significant lag time (using a WhatsApp video link). However, the AI tracking is based on facial recognition tracking, which creates challenges in keeping the patient in the full field of view. Additionally, it was not possible in any position to see below the patient’s knees. For this reason, it was not possible to safely conduct the timed Up & Go test, Berg Balance Test, or sit-to-stand test. Faster walking speeds also resulted in the patient leaving the field of view momentarily. The TV portal is confined to a TV room, which may make walking assessments challenging.

#### Safety

Concerns exist around using Facebook products and services regarding personal data [18,19]. Over the last decade, Facebook has received numerous fines for their mishandling of user data [20]. Facebook’s business model is based on their use of data [21], and their pixel software allows tracking of users across the internet even if they have not logged into a Facebook service. However, current NHS policy on use of Facebook platforms such as WhatsApp (used for Facebook Portal TV) is that “It is fine to use...to communicate with colleagues and patients/service users...where there is no practical alternative and the benefits outweigh the risk” [22].

### Mobile Phone and a Friend or Carer

#### Efficacy (Assessment Challenge)

Use of WhatsApp with another person holding the camera enabled the clinicians to undertake a complete assessment using all 5 outcome measures. With clear instructions from the clinician, the friend was able to offer multiple fields of view of the patient.

#### Delivery

A cost may be associated with the friend or carer being at the patient’s house.

#### Safety

There are some safely considerations related to the assisting friend bumping into or tripping over furniture while tracking the patient with the camera rather than watching where they are going. Data security was identified as an issue, although guidance from the NHS’s digital health technology unit, NHSX, seems to be more liberal given the COVID-19 pandemic [23,24].

### Mobile Phone and a Flexible Hose Stand

#### Efficacy (Assessment Challenge)

The flexible hose allows the patient to be guided by the clinician to achieve the appropriate field of view. At a distance of 3 m, the patient is in full view of the camera, and it was possible to complete all 5 assessment challenges.

### What Type of Consultation or Patient Group Would These Technologies Be Useful For?

The spectrum of “technology readiness” of the patient and their relative or friend is critical in determining suitable options. For a patient with no smartphone, no Wi-Fi access, and no relatives or friends using such technologies, video consultations that require assessment of movement would be inaccessible. This would not be the case for a digitally well-connected patient. The clinical condition will also pose specific challenges, irrespective of the technology at hand [25]. We created some “hypothetical patients,” that is, “mental constructs” that we established by taking the technology use and skills, various disabilities and physical limitations, and other characteristics of family members of the authors and “mentally” combining these with typical clinical conditions encountered by the therapists in the team. Table 3 gives examples of these hypothetical patients and how the combination of technology and their clinical condition might affect their choice of technology.

**Table 3:** Technology options for patients at different levels of technology readiness and with different clinical presentations.

Hypothetical patient^a^	Clinical condition drawn from clinical experience	Likely choice of technology
The patient owns an iPad and has Wi-Fi access. The patient uses email and FaceTime but does not have a mobile phone, is nearly blind, and has very limited hearing. They are technologically dependent on family members to set up apps or maintain technology.	Frail, difficulties with balance when standing and walking, regular falls	Although this patient could participate in a FaceTime call, the camera angle would be difficult and setting up any new technology would be difficult; hence, a friend or family member with a smartphone would be the best option.
The patient regularly uses a laptop computer, Skype, and Facebook Portal TV (via smart TV), which they use to stay in contact with family. They also use a tablet and laptop computer and although they have and use a smartphone, they tend to use the larger “fixed” technologies. The patient lives in an isolated location and would not want to involve a family member or friend in the consultation.	Pain and stiffness in shoulder	Using technology the patient is used to, Facebook Portal TV (the clinician does not need to see the patient’s feet) would work well. The next option might be the wide-angle webcam, which would fit their laptop. Pivo as a third option would be possible but would take more time to set up.
Same person as above but with a different clinical condition.	Knee and ankle pain and stiffness; independent walking with mild unsteadiness	A wide-angle webcam would be the first choice, as the clinician needs to see the patient’s feet. Pivo would be the second option.
The patient has a smartphone, laptop computer, Facebook Portal TV, tablet, and Wi-Fi access, but sometimes struggles with technology. They live with a partner who is a technology enthusiast. They would be willing for their partner to help with the consultation.	Neck pain with poor posture	The first choice is the partner using a smartphone, as no setup or delivery of equipment is required. If the partner was not available, the patient would probably opt for a wide-angle webcam with a simple USB connection.
Same person as above but with a different clinical condition. Although a household Facebook TV portal is available, the patient struggles to use it.	Pelvic girdle pain	The patient may prefer not to share this consultation with their partner, but the partner would be able to set up the Facebook TV portal before leaving the room. If the partner is not available, a wide-angle webcam is the easiest “plug and play” option.
The patient is a “digital native” smartphone user living in accommodations with relatively limited space.	Gait problems as a result of multiple sclerosis	Pivo would be the first option, as the patient does not have a device for the easy plug and play option of a wide-angle webcam. A simple mobile stand might be a second option.

^a^The technology use of the hypothetical patients is based on that of family members of the therapists.

## Discussion

### Principal Results

The COVID-19 pandemic has resulted in rapid uptake of the use of video consultations; however, in physiotherapy and rehabilitation, this uptake has been hampered by the difficulty of assessing movement. We identified three main technology approaches to address this problem: various rotating devices, sometimes described as desktop robots (Kubi and Pivo), stationary lenses that are either wide-angled or track the focus (wide-angle webcams and Facebook Portal TV), use of simple mobile holders, or assistance from other people. We tested the use of these approaches with coauthors taking the role of either the clinician or patient, and then we applied our understanding to “hypothetical patients.” There is no “one size fits all” approach in the use of video consultations [26], and similarly, the interplay of technologies in place, patient confidence, skills and support, and clinical conditions will determine the best technology to support assessment of movement in a video consultation. In relation to older people using assistive technologies, Greenhalgh et al [27] described the idea of bricolage, pragmatic customization, and combination of devices by the participant or friend or family. The same idea of “whatever works” applies to video consultations involving assessment of movement.

The two mechanical tracking devices, Kubi and Pivo, had significant differences in cost (Kubi £437 [US $600], Pivo £85 [US $118]). Our experience indicated that the Kubi would be of use when clinicians can move the camera angle to obtain a good angle when movement takes place within that new field of view; however, it proved difficult to effectively track movement, mainly because of the speed of response of the device. Kubi, however, was viewed as useful for “looking around” the home environment to observe possible safety hazards, but it was considered expensive for that modest role. The Pivo is, in our opinion, the better device to mechanically track the patient’s movements, as it provided a rapid response and did so automatically. Potential problems that we experienced with Pivo were that the Pivo Meet software required the patient to contact the clinician (rather than the clinician initiating contact with the patient) and that tracking was only enabled in the horizontal plane. Also, the video call must be conducted using the Pivo Meet software, whereas Kubi uses parallel software to control movement of the device while the video call can still be conducted using the service provider’s preferred video call software. Both devices require further work to develop easy patient setup routines and to test these with real patients.

Based on our experience, the use of a wide-angle webcam is likely to be the easiest to set up for patients who have a laptop or desktop computer. Facebook Portal TV, for those patients who already have it installed, could play a useful role provided that the clinician does not need to see the patient’s feet. The technologically simplest approach is to get another person to use a mobile phone to “film” the patient during the videoconference, but not every patient has access to another person, and they may not wish to share their consultation for reasons of confidentiality and privacy. The possible addition of a simple adjustable stand (£25, US $35) may be sufficient to enable patients to angle their phones or tablets to enable the clinician to have a better view of the movement.

An internet-based goniometer has demonstrated good to high validity and reliability of telerehabilitation in orthopedics and stroke when assessing joint range of motion of upper limb joints [28-32]. Similarly, adaptation of existing movement sensor technology (such as the Microsoft Kinect [33]) or other apps such as Coach’s Eye [34] could improve the accuracy of recording of joint range. However, these adaptations risk adding further layers of complexity to an already technologically challenging scenario. Other simple devices include a large “paper protractor” or asking permission from the patient to take a screenshot in order to use a program such as Microsoft Paint or Adobe Photoshop to perform goniometric calculations. However, processing these data accurately requires awareness of, and compensation for, issues such as parallax. Given the increased awareness of the need for effective remote monitoring systems, data sets are being gathered to address these challenges, meaning that this is an area that is likely to develop significantly moving forward [35].

The practical barriers of using devices such as Pivo or a wide-angle webcam may be related to the delivery and retrieval by the health provider and to the setup by the patient. Some health informatics services, such as those supporting people with chronic obstructive pulmonary disease at home, have been successfully using courier services to deliver simple-to-install equipment and have then been providing telephone support to patients in setting up this equipment (R Jones, personal communication). Further exploration of the feasibility and long-term cost of delivering and collecting different technologies by courier is needed.

Across all technologies, clear setup instructions are required, ideally coproduced with service users, and available in different formats, such as paper, electronic, or instructional videos. Setup has been an issue with normal video consultations [36], and our experience of the included instructions for Kubi, Facebook Portal TV, and other devices was that they were not as “usable” as they need to be. Instructions for clinicians and patients need to be professional in appearance, concise, and clear, with a comprehensive step-by-step guide, including for software installation. Also included should be a troubleshooting section, such as what to do if the Wi-Fi is switched off and how to increase the volume. Establishing the right settings and options for both clinician and patient is critical; hence, there is a need for inclusion of this information in the user instructions. If a user instruction video is produced, it should be subtitled (for people with hearing impairment), but there should also be a written guide. If the NHS becomes a major purchaser of such technologies, it could use its purchasing power to encourage manufacturers to produce easier and better-explained technology setups.

Consideration of the technology selected and the confidence of the user is particularly important and, where possible, there is a need to “bootstrap” from the known technologies in use by an individual. It is particularly important to consider issues such as cognition, anxiety, and sensory impairments such as vision or hearing. Once the clinician is experienced, they should also be able to give assistance over the telephone if the patient is struggling with setup. Alternatively, students or other community support organizations (eg, in Cornwall, the Cornwall Rural Community Charity [37], or nationally, the Good Things Foundation [38] or Digital Eagles [39], may be able to support patients using their existing technologies.

Outpatient consultations were resumed during the COVID-19 pandemic for many but not all services, and not to all patients. A risk/benefit judgement was made if patients were highly vulnerable or shielding. Conducting face-to-face consultations was feasible following the latest guidance on infection prevention and control with full personal protective equipment. However, this approach does not eliminate all risks. Patient choice is central to this decision. Face-to-face consultations can be difficult for many patients who have trouble travelling to the local hospital owing to both feasibility and cost issues.

Getting a family member or friend to hold the mobile phone or tablet for the video consultation, ensuring that the patient is “in shot,” may be more reliable than using these technologies and may be safer as well. There is evidence from our national survey [5] that this is currently occurring: “Our clients generally do not carry out video consultations on their own, they would normally have some support from a carer or family member” (Occupational Therapist, Neurology). This approach provides some advantages with regard to safety, although it does not completely resolve the issue when standby or hands-on assistance is needed (for example, with a standing balance task) given the requirement to hold the device. This approach is also problematic if the friend of family member is requested to assist in moving the limbs of a patient at the same time they hold the camera. Another disadvantage is that such assistance is likely to be required at each consultation; in contrast, technological alternatives might enable greater patient autonomy and privacy. In our survey [4], practitioners reported that family members provided a number of different types of support in video consultations, including technical support (setting up the technology, positioning the camera), physical support (helping to move or guide the patient, standby assistance for safety), and psychological support (reassuring the patient, clarifying instructions). However, this is not without difficulties: “It can be hard for patients/family members to get the right technique [for positioning the camera]” (Physiotherapist, Musculoskeletal/Rheumatology).

Technology is advancing rapidly. Two lines of current research that may help in assessing movement remotely are use of patient-wearable technologies [6,40] and more intuitive clinician interfaces, including use of wearable headsets [41]. Aggarwal explored the use of “smart socks” [42], which may be able to transmit data about foot pressures and balance to clinicians, and such technologies may prove to be useful additions to improve visual data.

The technology that is sometimes used in addition to technology for video consultations may raise concerns about data privacy and security for both patients and clinicians. This is a rapidly changing environment both for the technology and advice given by relevant bodies. For example, we originally thought that Facebook Portal TV was unlikely to receive approval for use by NHS trusts because of data security concerns; however, views on balancing data security concerns versus access during the pandemic indicate that opinions seem to be shifting. Advice from the NHSX Information Governance team [23,24] states that it is acceptable to use video conferencing tools such as Skype, WhatsApp, and FaceTime as well as commercial products designed specifically for this purpose, particularly as a short-term measure. Although NHSX states that any video consulting tool can be used provided there has been an appropriate local risk assessment [24] and, for example, Healthwatch seems to assume that Zoom may be used [43], some trusts still restrict use of video consultations to Microsoft Teams or Attend Anywhere.

The most promising approaches that we explored were use of wide-angle webcams, Pivo, a simple stand to hold a mobile phone, and obtaining help from another person with a mobile phone. Further testing and observational studies with patients within a clinical context are now needed. Equipment loans are integral to standard NHS practice; hence, it is appropriate to explore whether this should be extended to the loan of assistive devices to enhance the effectiveness of video consultations.

### Limitations

Our study was a preliminary exploration of currently available technologies with the use of role-play by clinicians. Technology development is rapid; by the time of publication, the devices reviewed may have progressed significantly, and new devices may have become available. However, our study provides guidance on potentially productive lines of inquiry and further research. Our exploratory study has been conducted by just one team, and further work by others would help validate our approach and conclusions. Furthermore, our work was carried in the United Kingdom; these results may not easily be generalized to resource-limited environments and developing countries.

### Comparison With Prior Work

We were only aware of one previous study of devices to assess movement in video consultations. Wu et al’s study [10] of 5 older people with self-reported mobility impairments reported limitations of using the Kubi device but did not investigate other technologies**.**

### Conclusions

Our findings suggest that the “technology readiness” of the patient and clinician, the clinical condition, and the availability of support from another person are important factors to consider when implementing technologies, such as those we have reviewed, to remotely assess movement as part of video consultations. The most promising off-the-shelf approaches seem to be use of wide-angle webcams, Pivo, a simple mobile phone holder, and obtaining help from another person with a mobile. Comparative clinical trials of these approaches, perhaps in the form of a preference trial, would be worthwhile.

## Appendix

Multimedia Appendix 1 Literature search details.

Multimedia Appendix 2 Hardware specifications at the time of the study.

Multimedia Appendix 3 Notes on other devices that were not tested.

Multimedia Appendix 4 Clinical instructions for the technology assessment.

## Abbreviations

AI: artificial intelligence
EPIC: eHealth Productivity and Innovation in Cornwall
NHS: National Health Service
POMA-G: Tinetti Performance-Oriented Mobility Assessment gait scale
