# Supplementary material for: Technologies to Support Assessment of Movement During Video Consultations: Exploratory Study
Source: JMIRx Med. 2021 Sep 24;2(3):e30233. doi: 10.2196/30233 (PMC10414296; doi:10.2196/30233)

**APPENDIX 1**

**LITERATURE SEARCH**

A literature review was conducted to explore the following question:

“How effective is technology to assess movement and mobility remotely”.

A broad search for relevant search terms was conducted in 3 large databases: Web of Science, CINAHL and MEDLINE. Of the refined search, articles were identified using combined search terms. A date limiter of 2017-2021 was used to reflect relevant and up to date use of technology. The reason for starting with 2017 is that:

(i) Routine use of video calls in clinical consultations is relatively recent. Starting with a very simple search of Web of Science on video consultations gave 2465 results, half of which were from 2017 onwards. But if the search is changed to video consultation AND physiotherapy Web of Science only returned 21 results - all but one of which were 2017 onwards.

(ii) Kubi was introduced to the market in 2012. It was likely that any study making use of it in clinical video consultations was not going to reach press until 2015 at the earliest.

(iii) As we were also searching via Google and had had a ‘watching brief’ on technology developments related to telepresence robots over the last decade we thought a five-year review of the literature was adequate.

The search was also limited to full, available English articles with an advanced search in Web of Science (nursing, general medicine, cardiac care, rehabilitation, health care services and pediatric categories) as the application of search results across all categories was beyond the scope of this evaluation.

On evaluation articles were included that utilised video-based technology to assess movement in remote settings. Video consultations without physical assessments, irrelevant technology or non-movement related assessments and articles that did not consider the effectiveness of the technology were excluded.

**Search Strategy Terms**

The below search strategy uses a logic grid and is based on the PCC framework. A specific population was not identified in the search as movement-based examinations can occur in diagnosed and undiagnosed populations.

| P (Population) | N/A |
| --- | --- |
| C (Concept) | Technology used to support movement assessment during video consultations |
| C (Context) | Video consultations in physiotherapy |

A movement and mobility related concept in a physiotherapy related context was used as opposed to healthcare in general as the ‘healthcare’ context resulted in a very wide scope of application beyond the needs of this evaluation.

| ***Population*** | ***Concept 1*** | ***Concept 2*** | ***Context 1*** | ***Context 2*** |
| --- | --- | --- | --- | --- |
| N/A | Device | Mov* | Tele* | Consultation |
|  | Tech* | Mobility | Virtual | mHealth |
|  | Tool | Physical* | Online | eHealth |
|  | Equipment | Gait | Video | Appointment |
|  | Machine | Walking | Digital | Service |
|  | System | Balance | Record* | Session |
|  |  | Motor |  | Intervention |
|  |  | Strength |  | Treatment |
|  |  | Power |  | Physiotherapy |
|  |  | Tone |  | Physical Therapy |
|  |  | Range |  | Physical therapist |
|  |  | Contracture |  | Physiotherapist |
|  |  | Fatigue |  |  |
|  |  | Stamina |  |  |
|  |  | Dexterity |  |  |

Search term strategy:

1. Each column was searched individually, e.g., physical condition OR physical disability OR physical impairment.
2. All individual searches based on each column were combined with AND criteria.
3. The combined AND search strategy proceeded to include context 1 and 2, and concept 1 and 2.
4. Date limiter: 2017-2021, full text, English articles with advanced search settings of nursing, general medicine, cardiac care, rehabilitation, health care service and pediatric categories in Web of Science.
5. Results from the AND combined searches were reviewed for relevance for this evaluation.

Results as of May 2021.

| ID | Search terms | Web of Science | Medline | Cinahl |
| --- | --- | --- | --- | --- |
| 1 | Device OR Tech* OR Tool OR Equipment OR Machine OR System | 4,227,249 | 62,454 | 52,369 |
| 2 | Movement OR Mobility OR Physical* OR gait OR walking OR balance OR motor OR strength OR Tone OR range or Contracture OR fatigue OR stamina OR dexterity OR power | 3,007,723 | 34,041 | 32,379 |
| 3 | Tele* OR Virtual OR online OR video OR digital OR record* | 1,064,874 | 18,173 | 22,742 |
| 4 | Consultation OR mhealth OR eHealth OR appointment OR service OR session OR intervention OR treatment physiotherapy or physical therapy or physiotherapist or physical therapist | 928,941 | 31,188 | 47,441 |
| 5 | #1 AND #2 AND #3 AND #4 | 2036 | 668 | 726 |


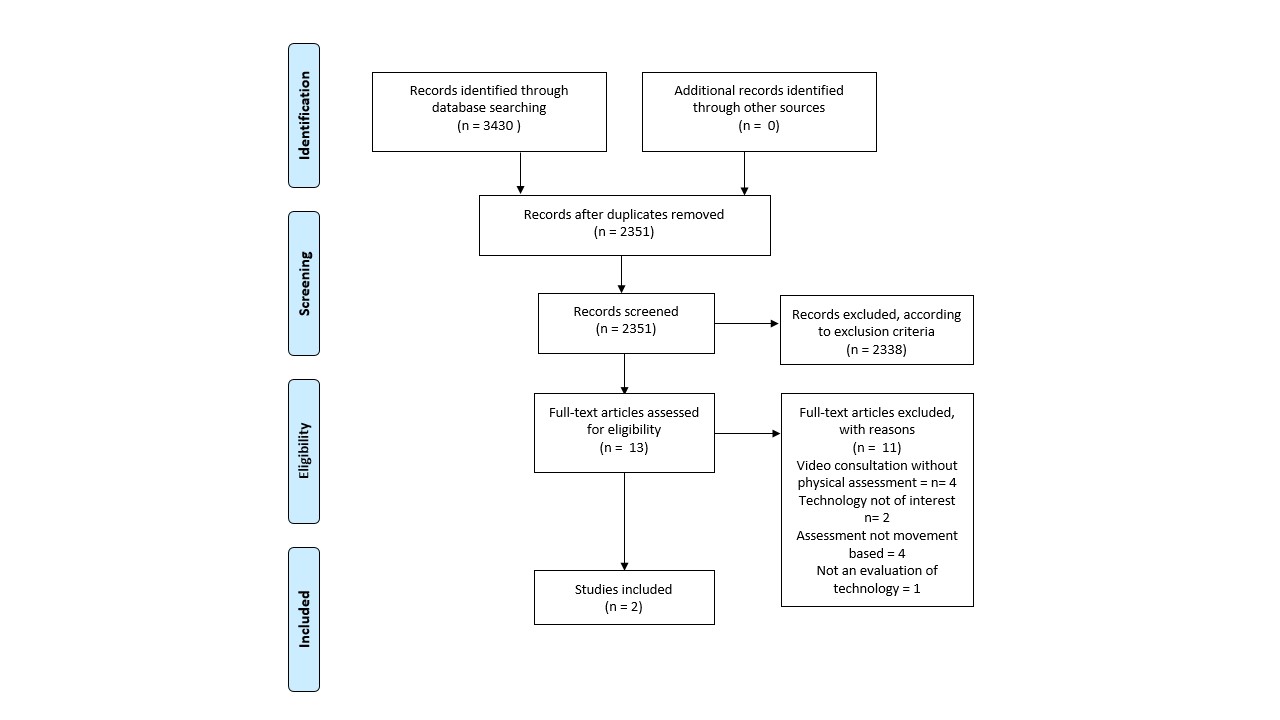

Supplement: Multimedia Appendix 1 [file xmed_v2i3e30233_app1.docx]
