# Supplementary material for: Technologies to Support Assessment of Movement During Video Consultations: Exploratory Study
Source: JMIRx Med. 2021 Sep 24;2(3):e30233. doi: 10.2196/30233 (PMC10414296; doi:10.2196/30233)
Supplement: Multimedia Appendix 2 [file xmed_v2i3e30233_app2.docx]

**Technologies to support video-consultations assessing movement: exploratory study**

**APPENDIX 2**

**HARDWARE SPECIFICATIONS AT TIME OF STUDY**

**Kubi Plus:** desktop robotics that can be remotely controlled by clinician during appointment. For 10 to 13 inch tablets, 300° Pan, +/- 45° Tilt, Weight with Base 370g, 6.4cm Width without base and 32.39cm Height without base. The Kubi Connect App is available on iPhone and iPod touch running iOS 9.0 or later; iPad running iPadOS 9.0 or later; Mac with Apple M1 chip running macOS 11.0 or later; Android 4.3 and later. The Kubi Connect Widget V1.2 for Windows: used on Windows 10.

<https://www.kubiconnect.com/e-commerce/kubi-plus.html> (Last accessed 29/3/2021).

**Lenovo Tablet:** (Processor: Qualcomm® Snapdragon™ 212; Display: 10.1” HD (1280 x 800) IPS display; Audio: 2 x front-facing speakers with Dolby Atmos®; Memory: 1GB/2GB/3GB RAM; 16GB/32GB ROM; Up to 128 GB microSD card; Dimensions(W x D x H): 247 mm x 171 mm x 8.9 mm; Weight: 522g; Battery: Up to 13 hours; Camera: Rear: 5 MP autofocus; Front: 2 MP fixed focus: Connectivity: WiFi 802.11 b/g/n, 2.4 GHz; Bluetooth 4.0; Ports: USB 2.0; 3.5mm audio jack. <https://www.lenovo.com/gb/en/tablets/android-tablets/lenovo-tab-series/Lenovo-Tab-E10/p/ZZITZTATB9X> )

**Pivo pod:** Desktop panoramic mobile mount (with bubble level), extendable stabilizer legs, tripod ready (¼" UN thread), remote control, over internet control and 4 speed 4 sec/360° rotation. The Pivo Meet App is available on iPhone and iPod touch running iOS 13 or later and on Android devices running Android 8.0 or later. Mobile browsers: links sent from the app on both Apple and Android devices only worked when opened in browsers on mobile devices such as phones or tablets. Edge, Chrome and Firefox Desktop browsers run on Windows 10 desktop were unable to connect to the Pivo Meet call. <https://uk.getpivo.com/> (Last accessed 11//4/2021)

**Facebook TV Portal**: Smart video calling for TVs with automatic pan and zoom 12.5 MP, - 120° Field of View camera. Works with; Messenger, Whatsapp. Connection via Wi-Fi and Bluetooth. Smart Sound enhances the voice of whoever is talking, while minimising background noise via 8 Mic Array. Alexa Built in. Mic and Camera disable button, integrated camera cover. Connects to TV via HDMI cable (not included). Colour: Black. Dimensions 317 x 190.5 x 571.5 mm (H x W x D), Weight 308 g. <https://portal.facebook.com/gb/> (Last accessed 11/4/2021)

**Wide Angle webcam:** Logitech Brio Stream Webcam, Ultra HD 4K Streaming Edition, 1080p/60fps Hyper-Fast Streaming, Wide Adjustable Field of View for Gaming, Works with Skype, Zoom, Xsplit, Youtube, PC/Xbox/Laptop. Connectivity technology USB; Lens type Zoom. Weight 90 g. <https://www.logitech.com/en-gb/product/brio-stream-4k-hd-webcam> (Last accessed 11/4/2021)

**Mobile Phone**: Several mobiles of co-authors were used. This description is included as typical. iPhone XR. 6.1-inch Liquid Retina display (LCD); 12MP camera with OIS and 7MP TrueDepth front camera—Portrait mode, Portrait Lighting, Depth Control, and Smart HDR; IoS 13, Screen 6.1 inch.4 grams.

**Broadband speeds**: Trials took place in various locations and various broadband speeds were achieved, typically, download speeds of 100-380 Mbps and upload speeds of 8-30 Mbps.
