# Supplementary material for: Technologies to Support Assessment of Movement During Video Consultations: Exploratory Study
Source: JMIRx Med. 2021 Sep 24;2(3):e30233. doi: 10.2196/30233 (PMC10414296; doi:10.2196/30233)
Supplement: Multimedia Appendix 3 [file xmed_v2i3e30233_app3.docx]

**Technologies to support video-consultations assessing movement: exploratory study**

**APPENDIX 3**

**Notes on other devices not tested**

| [Aver CAM340+](https://communication.aver.com/model/cam340plus) £399  USB connection. Zoom, Microsoft teams, skype compatible. Windows, Mac, Android compatible. Small size (0.251KG).  Includes quick start guide. Monitor mount, table top application or has a ¼” tripod mounting screw. 120^O^ field of view  4x zoom with pan and tilt. PTZApp software for remote control of camera. 4K video quality. White balance for improved image quality. Built in Microphone, Plug and Play installation. Technical support available. |
| --- |
| [ZasLuke Intelligent tracking Camera](https://www.amazon.co.uk/ZasLuke-Intelligent-Adjustable-Microphone-Conferencing/dp/B08L6NRSTC/ref=sr_1_13?dchild=1&keywords=zasluke+wide+angle&qid=1614097661&s=computers&sr=1-13) £40.99  USB connection. Compatible with zoom, facetime, skype, etc 0.24KG. Height adjustable tripod. Webcam moves with you to 270o horizontally and 37o vertically. 110 degrees field of view. Caveat. Product description states: do not move too fast! How fast is too fast? Built in Microphone 1080p quality camera. Auto focus 80-300cm. Plug and Play. Switch between tracking and non-tracking by pressing the button on the rear (the flashing blue light indicates tracking mode on) |
| [OSBOT AI powered webcam](https://www.amazon.co.uk/OBSBOT-Tiny-AI-Powered-Auto-Frame-Auto-Exposure/dp/B08MWDVZBW/ref=sr_1_1?dchild=1&keywords=AI+tracking+camera&qid=1614098760&s=computers&sr=1-1) £239  Small size 0.59KG, Windows/mac compatible, Zoom, Facetime, teams etc compatible. USB. Operates with lower user bandwidth. PTZ app software for basic camera functions such as movement and zoom. AI tracking capability with autofocus. 2x zoom capability. 150 degree field and 45 degree tilt. Maximum speed 120degree/second. White balance for better video quality. 1080p video resolution. Built in microphone. Plug and play instillation. Technical support via email |
| [Meeting OWL](https://uk-shop.owllabs.com/products/meeting-owl-pro?utm_source=adwords&utm_campaign=UK_Non-Brand_Core&utm_medium=ppc&utm_term=%2Btracking%20%2Bconference%20%2Bcamera&hsa_kw=%2Btracking%20%2Bconference%20%2Bcamera&hsa_mt=b&hsa_tgt=kwd-934310377134&hsa_src=g&hsa_ad=454950643429&hsa_ver=3&hsa_cam=10782728111&hsa_net=adwords&hsa_acc=2493962266&hsa_grp=109766536201&gclid=CjwKCAiAyc2BBhAaEiwA44-wW9j8ZajNOVanxeCmamuUeXSR06YZPZMEbG57LZlBB6D6WdSau-4FTRoCo0MQAvD_BwE) £799  Integrates with Zoom, hangouts, meet etc, Quick start guide. Described as easy user set up. 360 degree field of view. Built in microphone and speaker. 720p camera (1080p camera available in the Pro version £999). Has the potential to link in multiple Owl’s for multi-participant meetings. Plug and play set up. Technical support provided by Owl and replacement policy (cost £149) |
| New View [Wide-angle conference camera](https://www.amazon.co.uk/Conference-Microphone-Speaker-Desktop-NV-VA101/dp/B08SMM5WRF/ref=sr_1_1_sspa?dchild=1&keywords=wide+angle+webcam&qid=1614783701&refinements=p_36%3A428447031&rnid=428432031&s=computers&sr=1-1-spons&psc=1&spLa=ZW5jcnlwdGVkUXVhbGlmaWVyPUEzVFI1UjIyQ1gwTFhUJmVuY3J5cHRlZElkPUEwODg3Nzk0QlNRMUtHOEswOUlFJmVuY3J5cHRlZEFkSWQ9QTAwNDkyMjcyWEMwNENVWFJFSjI2JndpZGdldE5hbWU9c3BfYXRmJmFjdGlvbj1jbGlja1JlZGlyZWN0JmRvTm90TG9nQ2xpY2s9dHJ1ZQ==) £138  Compatible with zoom, skype etc, 0.5kg, Sits on laptop or tripod, Requires patient to have a laptop. 105 degree field of vision Built in camera and speaker. 1080dp camera quality. Plug and Play |
