# Supplementary material for: Technologies to Support Assessment of Movement During Video Consultations: Exploratory Study
Source: JMIRx Med. 2021 Sep 24;2(3):e30233. doi: 10.2196/30233 (PMC10414296; doi:10.2196/30233)
Supplement: Multimedia Appendix 4 [file xmed_v2i3e30233_app4.docx]

**Technologies to support video-consultations assessing movement: exploratory study**

**APPENDIX 4**

**CLINICAL INSTRUCTIONS FOR TECHNOLOGY ASSESSMENT**

1. **Sit to stand in 30 seconds:**

Equipment needed: 17-inch high chair with back, stopwatch, and wall space. The 30-second chair test is administered using a folding chair without arms, with seat height of 17 inches (43.2 cm). The chair, with rubber tips on the legs is placed against a wall to prevent it from moving. The participant is seated in the middle of the chair, back straight; feet approximately shoulder width apart and placed on the floor at an angle slightly back from the knees, with one foot slightly in front of the other t help maintain balance. Arms are crossed at the wrists and held against the chest. Demonstrate the task both slowly and quickly. Have the patient practice a repetition or 2 before completing the test. If a patient must use their arms to complete the test they are scored 0. The participant is encouraged to complete as many full stands as possible within 30 seconds. The participant is instructed to fully sit between each stand. While monitoring the participant’s performance to ensure proper form, the tester silently counts the completion of each correct stand. The score is the total number of stands within 30 seconds (more than halfway up at the end of the 30 seconds counts as a full stand). Incorrectly executed stands are not counted. The number of stands a person can complete in 30 seconds is recorded.

1. **Timed Up and Go Test**

Equipment needed: stop watch, standard height chair with armrests, measuring tape, tape, cone. Time to administer less than 3 minutes. No training required. Individuals are given verbal instructions to stand up from a chair, walk 3 meters as quickly and safely as possible, cross a line marked on the floor, turn around, walk back and sit down. The test includes the time the individual takes to get out of the chair after s/he is told to go. Individuals are permitted to use the assistive device they typically use in the community, but without the assistance of another person.

1. **Berg Balance Test**

Equipment required stopwatch, standard height chair (18-20 inches with armrests), standard height chair without armrests, step or stool of average height (7.75-9 inch step stool), ruler, slipper or shoe. 15-20 minutes to administer. Fourteen items include static and dynamic activities of varying difficulty. Item-level scores range from 0-4 determined by ability to perform the assess activity. Item scores are then summed. Maximum score=56.

1. **Lower limb Range of Motion at ankle:**

Estimated range of motion at ankle based on visual inspection when the patient is in a seated position, dorsiflexion, plantarflexion, inversion, eversion.

1. **Upper limb Range of Motion at shoulder joint:**

Estimated range of motion at shoulder based on visual inspection when the patient is in a seated position: forward flexion, abduction, internal rotation, external rotation.
